# Being vulnerable with viewers: Exploring how medical YouTubers communicated about COVID-19 with the public

**DOI:** 10.1371/journal.pone.0313857

**Published:** 2024-12-20

**Authors:** Seung Woo Chae, Noriko Hara, Harshit Rakesh Shiroiya, Janice Chen, Ellen Ogihara

**Affiliations:** 1 Department of Journalism and Creative Media Industries, College of Media and Communication, Texas Tech University, Lubbock, Texas, United States of America; 2 The Luddy School of Informatics, Computing, and Engineering, Indiana University, Bloomington, Indiana, United States of America; 3 Medpace, Cincinnati, Ohio, United States of America; 4 Department of Psychological and Brain Sciences in the College of Arts & Sciences, Indiana University, Bloomington, Indiana, United States of America; 5 Sporecyte, Orem, Utah, United States of America; Universidad Internacional de La Rioja, SPAIN

## Abstract

This study explores COVID-19 communication between medical experts who upload YouTube videos related to health/medicine (hereinafter medical YouTubers) and their viewers. We investigated three specific elements: (1) how medical YouTubers’ use of words related to analytical thinking is associated with their viewers’ engagement, (2) how medical YouTubers’ use of different types of emotion is associated with their viewers’ engagement, and (3) the emotional alignment between medical YouTubers and their viewers. We collected 194 COVID-related video transcripts from five YouTube channels and 375,284 comments from those videos. We employed natural language processing to analyze the linguistic and emotional dimensions of these two text sets including analytical thinking, positive emotion, and negative emotion, the last of which was divided into anxiety, anger, and sadness. Additionally, three metrics provided by YouTube—the number of views, likes, and comments—were used as proxies representing user engagement. Our regression analysis results displayed that the medical YouTubers’ analytical thinking was positively associated with the number of views. Regarding emotion, anxiety was positively correlated with the number of likes and comments, while both positive emotion and anger were negatively associated with the number of views. Finally, both positive and negative emotions of medical YouTubers were found to be positively correlated with the corresponding emotions of their viewers. Theoretical and practical implications of these findings are discussed within the context of COVID-19.

## Introduction

Traditional methods of information dissemination and broadcast are rapidly evolving alongside new technology, with social media becoming a major scientific-information source [1]. Today, scientists should consider adapting to this new communication framework to share their findings more broadly [2]. During the COVID-19 pandemic, more non-experts engaged with scientists on social media due to their need for COVID-related scientific information [3]. Public health scientists and medical experts actively employed social media to combat COVID-related misinformation, including misinformation about mask-wearing and vaccines [4]. Though many scientists may still view social media as an optional knowledge-sharing tool, it is now a go-to source for non-experts seeking scientific information, particularly for younger generations [5]. Thus, it is helpful for scientists to be well-equipped with social media strategies when sharing their knowledge.

This study considers the question, “how can scientists best utilize social media to share scientific information with the public,” to be crucial to science communication, since a major area of science communication research is about connecting with a broader audience [6]. Recent literature has shared valuable findings on scientists’ general social media strategies (e.g., [7,8]); this study expands on this literature by investigating COVID-related communication, specifically between medical experts who upload YouTube videos related to health/medicine (hereinafter “medical YouTubers”) and their viewers. Particularly, main interests include: “How is medical YouTubers’ language associated with their viewers’ engagement to the video?” and “Is there emotional alignment between medical YouTubers and their viewers?” Based on these investigations, our ultimate objective is to provide practical suggestions for scientists to communicate more effectively with the public on social media, especially by using videos.

It is worth clarifying here that this study does not focus solely on medical YouTubers’ messages; while videos are essential to this study, we consider viewers’ comments just as important. Brossard [9] notes that the role of non-experts’ participation in online environments has changed the nature of science communication, creating new opportunities for two-way communication, i.e., public engagement with science [10]. Recent technological developments allow non-experts to communicate directly with scientists, without intermediaries such as journalists [11]. There is a need to research not just communication from scientists to the public, but also communication from the public to scientists in online environments [9,12]; some studies have examined this bidirectional communication (e.g., [13,14]). However, the current study particularly contributes to the research focusing on how medical YouTubers’ use of language relates to the interactions between them and their viewers.

## Literature review

### Social media communication strategies for scientists

Even before the emergence of COVID-19, literature has investigated social media as a tool for health/science communication in a variety of different health topics and contexts, albeit preliminary in nature. We share the salient characteristics of the literature here. First, much of the social media usage explored was that of health organizations, such as the CDC [15], Malaysia’s Ministry of Health [16], and the National Cancer Institute [17]. Guidry et al. [15] investigated strategic social media usage from the CDC, WHO, and Doctors Without Borders on Twitter and Instagram; they found the latter platform to be particularly valuable for social media communication. In contrast, Keller et al. [18] found that only a small number of public health professionals utilized social media platforms, such as YouTube and Facebook. Second, since younger generations were the major users of social media at that time, some suggested that social media could be used to better communicate with those who are in their teens and 20s. For example, communicating with and educating youth using social media regarding prescription opioid use [19] as well as warning young adults about tobacco and substance use with social media [20] were recommended. Lastly, one noteworthy suggestion in the literature was the presence of imagery. For instance, the inclusion of imagery was a strong predictor of likes and shares in a study of Facebook posts about diabetes [21].

It is worth noting one particular limitation of the literature before COVID-19: science communication research on the use of social media tended to focus on information dissemination rather than interactional engagement [18]. Especially with video posts, most studies were limited to one-way communication. Notably, studies of educational use of science-related YouTube videos, such as those about synthetic biology education [22] or renewable technology videos [23], were focused on the YouTubers’ messages, not the messages from the viewers (i.e., comments).

As the COVID-19 virus became a concern in late 2019, scientists began to more actively communicate with the public about science via social media, and their communication strategies have been spotlighted in academia. Some studies on science communication argue that productive science communication is founded upon trust between scientists and non-experts [24]. To this end, scientists’ communication should incorporate a story, utilize emotions, exhibit vulnerability, empower audiences, demonstrate significance, and employ humor [2,25]. These practices garner trust, engagement, comprehension, and interest from audiences, leading to a better-informed and better-off public [24,26].

Of late, storytelling has been a communication strategy regularly suggested by organizations and researchers [e.g., 27,28] as a way to transform a heavy presentation into narratives. Traditionally, scientists delivered scientific knowledge in a logical, but often complicated, language, which tended to be abstract and context-free, so that findings could be generalizable [29]. However, recent studies of science communication suggest that scientists should consider delivering their findings with narratives, rather than with detailed explanations [29,30]. The beneficial characteristics of using narratives while learning science include emotionalization to make dry content more appealing, dramatization and personalization to help increase interest among learners, and fictionalization to guide construction of mental models [31]. Similarly, Dubovi and Tabak’s study [7] of science-related YouTube videos found that emotions are connected to both behavioral and cognitive engagement of users.

In this paper, we first address analytical thinking, as opposed to storytelling, and then focus on the emotionalization aspect. Finally, we investigate how medical YouTubers’ use of emotions in their videos are correlated with their viewers’ emotions via examining the emotional alignment between YouTubers’ transcripts and viewers’ comments.

### Analytical thinking in science communication

Before describing how analytical thinking is considered in science communication literature, we first wish to clarify what analytical thinking refers to in the present study to avoid any possible confusion. James Pennebaker, a pioneer researcher who has shed light on the relationship between human psychology and language [32,33], proposes analytical thinking as an important variable, which is “characterized by careful, effortful deliberation based on reason and logic” [34]. He and his research team suggest that this thinking style is expressed by a certain set of words (hereinafter “analytical words”), and Linguistic Inquiry and Word Count (LIWC), the natural language processing (NLP) software developed by the team, incorporates analytical thinking as a major variable [35]. Here, analytical thinking is conceptually opposed to narrative-oriented thinking; “people who are more analytic are more formal and detached, whereas those who are more narrative use language that is more personal and informal” [34]. In terms of both formality and emotional distance, communication based on analytical thinking and storytelling based on narratives contrast.

Existing literature is unclear about how the recommendation in the science communication literature—avoid analytical words and use storytelling/narratives [36]—is applicable to COVID-related communication, particularly in video, which is currently a very popular form of social media. To fill this gap, we examined medical experts’ videos on YouTube, the world’s most popular video-sharing social media platform [37]. This study examines how the inclusion of analytical words in the speeches of medical YouTubers in their COVID-related videos correlates with viewer engagement. In science communication literature, social media engagement is often measured with quantitative metrics including the number of views, likes, and comments [e.g., 7,38], which are possible proxies for representing viewers’ attention to, support for, and participation in the discussion section of the video, respectively. Therefore, this study tests how medical YouTubers’ analytical words in their COVID-related videos are associated with these three engagement metrics with the following research question:

*RQ1*: How are medical YouTubers’ analytical words associated with the numbers of views, likes, and comments in the discussion sections of their videos, respectively?

### Roles of emotion in science communication

As the use of emotion helps individuals’ meaning making [39], some researchers studied the use of emotions via humor [25,40] and emoticons [26] in science communication. As previous studies [41] indicated that scientists’ use of emotion in science communication would increase the audience engagement, literature on online user engagement encourages this “inclusion of emotion.” In [7] examining science-related YouTube videos and user engagement, the authors found that emotion increases viewers’ likelihood of leaving comments and triggers greater cognitive engagement. Similarly, Tatar et al.’s meta-analysis [42] of what makes online content popular found that triggering emotions within audiences is a strong predictor of popularity. Prior research further examined the relationship between emotion and the trustworthiness of information. For instance, Vosoughi et al. [43] detected emotion in text distributed via Twitter over ten years, to evaluate true and false information. They found that replies to posts containing true information triggered more sadness, anticipation, and trust. On the contrary, replies to false information posts include great surprises and disgust. Inspired by this study, Giachanou et al. [44] developed a model to use emotions as a way to identify false information.

Some studies attempted to particularly shed light on whether positive and/or negative emotions are important in science communication on social media. To illustrate, Djerf-Pierre et al. [45] found that expression of emotions was one of the main forms of high-level engagement in their qualitative analysis of popular science and journalism in YouTube videos about antimicrobial resistance. Tang et al.’s study [26] about scientists’ “Ask Me Anything” series on Reddit found that when a scientist applied positive social cues (such as humor, politeness, and comfort), participants did as well, leading to friendly, casual, and dynamic interactions. Other studies show that negative media had higher views and higher average ratings [46] as well as more likes [47]. Taken together, the findings about emotions in science communication are mixed.

Moreover, previous studies’ investigations are not necessarily limited to videos by scientists, but more broadly about videos related to scientific topics. The samples of YouTube videos examined included those created by a certain non-scientist group, such as journalists [45], as well as broader collections of YouTube videos gathered through keyword searches [46,47]. This means that the investigated samples were more inclusive than exclusive. Thus, the current study extends prior literature by examining the use of positive and negative emotions, particularly in the context of COVID-related YouTube videos uploaded by medical professionals. To that end, first, the following research question is proposed regarding positive emotion:

*RQ2*: How are medical YouTubers’ positive emotions associated with the numbers of views, likes, and comments in the discussion sections of their videos, respectively?

When it comes to negative emotion, three different types of negative emotion—anxiety, anger, and sadness—were explored in this study based on prior literature [48,49]. Some of the previous studies on social media users’ emotions during the COVID-19 pandemic tested specifically these three emotions. Ashokkumar and Pennebaker [48] examined how these three types of emotion had changed after the outbreak of COVID-19 by analyzing Reddit users’ text. Their results varied by the emotion type; anxiety and sadness increased after the outbreak, while anger dropped. Following this study, Chae and Lee [49] also addressed the same topic but in the context of a very distinct social media platform, Twitch, which supports live streaming. As a result, they found that anxiety and anger significantly increased after the WHO’s pandemic declaration, but sadness did not significantly change. These findings confirm the necessity of research that divides negative emotion into specific types of emotion rather than lumping them into a single blanket variable. Based on these previous studies, the current study examines the different types of negative emotion—anxiety, anger, and sadness—separately and sees their associations with viewer engagement, with the following three research questions:

*RQ3-1*: How is medical YouTubers’ anxiety associated with the numbers of views, likes, and comments in the discussion sections of their videos, respectively?
*RQ3-2*: How is medical YouTubers’ anger associated with the viewers’ numbers of views, likes, and comments in the discussion sections of their videos, respectively?
*RQ3-3*: How is medical YouTubers’ sadness associated with their viewers’ numbers of views, likes, and comments in the discussion sections of their videos, respectively?

### Emotional alignment between scientists and their audience

Prior literature on the new media environment explores emotional alignment between users as an important factor for their bonding. Döveling et al. [50] discuss the role of emotion in digital environments, especially on social media. They stated that emotional alignment helps bring individuals together and develop feelings of belonging to a group. In line with this conclusion, some research [2,26] found that effective science communication often involves establishing an emotional connection with the target audience. However, it is unclear how emotions expressed by scientists relate to the audience’s emotions. Previous studies examining sentiments of science-related YouTube videos focused on gender [13], comparison of pro-anorexia and anti-pro-anorexia [51], or certain types of technology, such as robots and AI [52].

Emotional alignment may occur through emotional contagion [53,54] as one’s emotions are influenced by others. Emotional contagion, if mindfully used, can be an effective tool for communication. On social media, emotions spread quickly, as Hill et al. [55] called it, “infectious.” Individuals shared various emotions on social media, especially during the COVID-19 pandemic [56,57]. Lu and Hong [56] shared the significance of negative emotional contagion during the pandemic on Chinese social media. Steinert [57] also found predominantly negative emotional contagion on social media during the pandemic and expressed concerns for its social influence. In a broader study of YouTube videos and channels, Rosenbusch et al. [58] examined both emotional contagion between YouTubers and viewers with individual videos and emotional homophily at the YouTube channel level. They found that both individual videos and channels influence viewers’ emotions, validating both emotional contagion and homophily.

While some studies have investigated the emotional aspects of COVID-related social media communication [e.g., 59], no studies have explored the emotional alignment as a result of contagion between scientists and their audiences. As such, we propose the following research questions to observe how medical YouTubers’ positive and negative emotions are associated with those of their viewers, respectively:

*RQ4-1*: How does medical YouTubers’ positive emotion associate with their viewers’?

*RQ4-2*: How does medical YouTubers’ negative emotion associate with their viewers’?

## Methods

### Sampling

To select medical YouTubers for our sample, three researchers searched for articles about popular medical and science YouTube channels using “medical YouTuber” and “science YouTuber” as initial keywords on Google and then expanded the search. As a result, we identified five directly relevant articles that recommend YouTubers discussing medicine, health, or science as their main topic. Based on these articles, our initial list of 21 medical YouTubers was built. Due to linguistic barriers, we excluded those whose videos were not in English. We also ruled out YouTubers without a license or degree to prove their medical expertise. Lastly, any channels with fewer than 10 COVID-related videos were excluded. After these steps, our final list included five YouTube channels (see Table 1). As of March 1, 2023, the average number of subscribers among our sample is 2.54 million users, ranging from 24,453 to 10.6 million users. The list is composed of four Doctors of Medicine (M.D.) and one Doctor of Osteopathic Medicine (D.O.). Three are from the U.S., and the other two are from the U.K.

**Table 1:** General information about medical YouTubers.

Channel Name	Number of Subscribers*	YouTuber’s Degree/License	YouTuber’s Country	Number of COVID-related Videos (*n* = 194)
**Doctor Mike**	10,628,681	Doctor of Osteopathic Medicine	U.S.	30 (15.5%)
**Doctor Mike Hansen**	1,069,173	Doctor of Medicine	U.S.	85 (43.8%)
**Medlife Crisis**	513,673	Doctor of Medicine	U.K.	14 (7.2%)
**Dr Hope’s Sick Notes**	480,947	Doctor of Medicine	U.K.	43 (22.2%)
**Medicine Deconstructed with Cedric Jamie Rutland MD**	24,453	Doctor of Medicine	U.S.	22 (11.3%)

*Note*. All the information including the number of subscribers was recorded on March 1, 2023.

We built our sample from COVID-related videos uploaded to these channels between 2020-January and 2023-January. Since this study focuses on COVID-related communication, we excluded videos that did not include at least one of the keywords—COVID, Corona, or SARS-CoV-2—in their title. Finally, we collected 194 COVID-related videos from the five channels. On average, these videos are 12 minutes and 10 seconds long, with 813,677 views, 26,607 likes, and 3,372 comments (including replies) as of March 1, 2023. This indicates that the videos received roughly one like every 31 views and one comment every 241 views.

### Data

We collected transcripts and comments, respectively, for each of the 194 videos. All of the 194 transcripts were downloaded via Downsub.com, which previous studies have used [e.g., 60]. For each channel, two transcripts were randomly selected, and one of the authors checked the quality of the selected transcripts by watching the corresponding videos. For comments, we initially collected all text in each comment section, including replies, via YouTube’s application programming interface (API). However, when analyzing positive and negative emotions of the comments, we decided to eliminate replies to other comments since the focus of our observation is on communication between medical YouTubers and their viewers, rather than communication amongst viewers. This exclusion left 375,284 comments. Of note: the unit of analysis of this study is the video, not the comment. Altogether, we collected 194 transcripts and 375,284 comments from the five channels.

From the transcripts, we measured the extent of analytical thinking, positive emotion, and negative emotion (anxiety, anger, and sadness) using the aforementioned NLP software LIWC, which has been used by many studies on social media [e.g., 49,61]. Furthermore, the three social media engagement metrics—the number of views, likes, and comments (including replies)—were recorded by checking the webpage of each YouTube video in our sample. To avoid any possible variance by time, the recording was completed within three hours. Of the 194 videos, three videos did not disclose their number of likes.

The current study is approved by the Institutional Review Board of Indiana University as exempt research. Additionally, as the collected data are from YouTube, this study abides by YouTube’s terms of service.

### Analysis

There are two analyses in this study. The first analysis addresses RQ1, RQ2, and RQ3, concerning medical YouTubers’ analytical thinking, positive emotion, anxiety, anger, and sadness, and was tested using negative binomial regression. The three metrics of engagement—the number of views, likes, and comments—are used as the dependent variables in this analysis. The second analysis addresses RQ4, which concerns emotional alignment between medical YouTubers and their viewers, and was tested with multiple linear regression.

#### Medical YouTubers’ language and viewers’ engagement (RQ1, RQ2, & RQ3)

In the first analysis, again, the independent variables—YouTubers’ *analytical thinking*, *positive emotion*, *anxiety*, *anger*, and *sadness*—were measured using LIWC. The NLP software was designed to measure people’s psychological states through their writing or speech [35]. Most of the variables in LIWC-2022, including positive emotion, anxiety, anger, and sadness, are computed by dividing the number of the words included in the category dictionary by the total number of words; put differently, each score represents the percentage of words related to the specific category. As exceptions, four categories—analytical thinking, clout, authenticity, and emotional tone—are summary variables, which are the only non-transparent categories in LIWC. According to the LIWC manuals [35,62], measurements for these four variables were derived from prior literature and converted into standardized percentiles based on LIWC’s data from large corpora.

Regarding the independent variables, we calculated the scores of the five LIWC categories—*analytical thinking*, *positive emotion*, *anxiety*, *anger*, and *sadness*—for each video’s transcript. As mentioned earlier, the LIWC-2022 manual describes the category *analytical thinking* as a metric of logical and formal thinking [35]. For example, words like “process” and “question” increase the analytical thinking score in LIWC. The same LIWC manual indicates that emotion-related variables including *positive emotion*, *anxiety*, *anger*, and *sadness* count when the word strongly implies the corresponding emotion. For instance, LIWC’s positive emotion, anxiety, anger, and sadness dictionaries include “happy,” “worry,” “hate,” and “cry,” respectively. When it comes to the dependent variables, the three engagement metrics—*number of views*, *number of likes*, and *number of comments* (including replies)—were used to represent the extent of viewers’ attention to, support for, and participation in the comment thread of the video.

We constructed three negative binomial regression models predicting each of the three dependent variables. Negative binomial regression was decided upon considering the overdispersion observed in all three dependent variables—*number of views* (*M* = 813,677, *SD* = 1,734,264), *number of likes* (*M* = 26,607, *SD* = 48,920), and *number of comments* (*M* = 3,372, *SD* = 5,348). Each of the three regression models has three control variables, including two in common. First, we included *number of days since upload* (*M* = 850, *SD* = 238) as a control variable in all models. This is because videos uploaded more recently tend to have lower numbers of views, likes, and comments, due to having less time since their release. Second, another control variable in common was *number of words in transcript* (*M* = 2,052, *SD* = 1,152), as it could significantly affect the influence of the individual words. Lastly, we controlled *number of subscribers* when using *number of views* as the dependent variable, while we controlled *number of views* when *number of likes* or *number of comments* was the dependent variable. Controlling the number of subscribers (*M* = 2,543,385, *SD* = 4,534,967) and views (*M* = 813,677, *SD* = 1,734,264) was essential given the high variances by channel or video. The scales of all control variables as well as *analytical thinking* were adjusted for ease of presentation (see details in the notes to Table 2). Lastly, the maximum number of iterations for the models regarding both *number of likes* and *number of comments* was increased to 100 to ensure the convergence of the fitting process.

**Table 2:** Negative binomial regression models for number of views, likes, and comments.

		N of views	N of likes	N of comments
		*B* (*SE*)	*B* (*SE*)	*B* (*SE*)
**Predictors**	Analytical thinking^a^	2.75*** (.58)	.29 (.47)	-.44 (.48)
	Positive emotion	-.11*** (.03)	-.02 (.27)	-.36 (.27)
	Anxiety	-.04 (.06)	1.29** (.46)	.94* (.47)
	Anger	-.38* (.19)	2.39 (1.58)	1.64 (1.61)
	Sadness	.16 (.17)	-.81 (1.35)	-.23 (1.38)
**Control variables**	Number of subscribers^b^	2.34*** (.28)		
	Number of views^c^		8.89*** (.44)	7.71*** (.45)
	Days since upload^d^	.86* (.42)	.24 (.35)	.08 (.37)
	Number of words in transcript^e^	.65 (9.08)	-8.84 (7.33)	-3.88 (7.55)
**Constant**		11.30*** (.51)	8.44*** (.41)	7.88*** (.42)

^a^The scale of *analytical thinking* was adjusted by dividing it by 100.

^b^The scale of *number of subscribers* was adjusted by dividing it by 10,000,000.

^c^When *number of views* was included as a control variable, its scale was adjusted by dividing it by 10,000,000.

^d^The scale of *days since upload* was adjusted by dividing it by 1,000.

^e^The scale of *number of words in transcript* was adjusted by dividing it by 100,000.

* *p* < .05

** *p* < .01

*** *p* < .001.

#### Emotional alignment between medical YouTubers and viewers (RQ4)

We examined the associations between the emotions of medical YouTubers and their viewers. As we divided RQ4-1 and RQ4-2, we measured positive and negative emotions separately. Using LIWC’s two variables, *positive emotion* and *negative emotion*, the medical YouTubers’ positive and negative emotions were measured from their transcripts. In the same way, we gauged viewers’ positive and negative emotions from their comments. Again, each score of LIWC’s *positive emotion* and *negative emotion* exhibits the percentages of words in the text showing the corresponding emotion. The LIWC scores were initially measured by comment (not comment thread). After this step, each video’s final score was computed by averaging the scores of all comments for the video. As mentioned earlier, replies were excluded in this particular analysis, given the scope of this study. Finally, we excluded 11 videos with fewer than 20 non-emoji-only comments, as the LIWC scores were significantly swayed by small numbers of comments; this left 183 videos for this analysis.

The linear regression model for RQ4-1 used medical YouTubers’ positive emotion as the independent variable, with viewers’ positive emotion as the dependent variable. The linear regression model for RQ4-2 used medical YouTubers’ negative emotion as the independent variable, and viewers’ negative emotion as the dependent variable. These regression analyses shared two control variables: *number of comments in the comment thread* (*M* = 2,050, *SD* = 3,336) and *number of words per comment* (*M* = 35, *SD* = 7). They were controlled, as they could potentially affect the results of both regression analyses. Additionally, in the regression analysis for positive emotion, the YouTuber’s opposite emotion, i.e., negative emotion, was included as a control variable given its possible influence. Likewise, the YouTuber’s positive emotion was controlled in the regression analysis for negative emotion.

## Results

### Descriptives

#### Social media engagement metrics

The average number of views across the 194 videos was 813,677 (*SD* = 1,734,264), ranging from 929 to 17,243,664 views. The average number of comments was 3,372 (*SD* = 5,348), ranging from 1 to 36,671 comments. Again, for the number of likes, we excluded three videos that did not disclose their like counts. With this exclusion, the average number of likes across the 191 videos was 26,607 (*SD* = 48,920), ranging from 47 to 318,665 likes.

#### Medical YouTubers’ language

The computations by LIWC displayed that the average score of *analytical thinking* in the medical YouTubers’ speeches, across the 194 videos, was 39.77 (*SD* = 18.52). Although this score is below the median percentile of the LIWC-2022 corpora, which include both written text and speeches, it is about 3.6 times higher than the average percentile of the *conversation* corpus in LIWC-2022 (*M* = 11.03, *SD* = 9.27). The average scores of *positive emotion* and *negative emotion* were .36 (*SD* = .31) and .43 (*SD* = .32), indicating that on average, 0.36% and 0.43% of all words spoken by the medical YouTubers were explicitly related to positive and negative emotions, respectively. When it comes to the specific types of negative emotion, the average scores of *anxiety*, *anger*, and *sadness* were .13 (*SD* = .18), .03 (*SD* = .05), and .03 (*SD* = .06), respectively. As with *positive emotion*, each of these scores represents the average percentage of the words that convey the corresponding emotion in the medical YouTubers’ speeches.

#### Viewers’ language

After excluding 11 videos with fewer than 20 non-emoji-only comments, the extent of positive emotion and negative emotion in viewers’ comments across the 183 videos were measured by LIWC. The average scores of *positive emotion* and *negative emotion* were 1.84 (*SD* = 1.00) and .83 (*SD* = .36), which indicates that on average, 1.84% and 0.83% of all words in the viewers’ comments are related to positive and negative emotions, respectively.

### Medical YouTubers’ language and viewers’ engagement

In the negative binomial regression analysis for “number of views” (see Table 2), *analytical thinking* was positively associated with *number of views* (*B* = 2.75, *SE* = .58, *p* < .001), while *positive emotion* (*B* = -.11, *SE* = .03, *p* < .001) and *anger* (*B* = -.38, *SE* = .19, *p* = .049) were negatively associated with it. The rest of the independent variables—*anxiety* and *sadness*—showed no significant association. Regarding the negative binomial regression analysis for “number of likes,” *anxiety* was positively associated with *number of likes* (*B* = 1.29, *SE* = .46, *p* = .005). All other independent variables did not have a significant association with the dependent variable. Lastly, the results of the negative binomial regression analysis for “number of comments” displayed that, again, *anxiety* was the only independent variable that was significantly associated with *number of comments* (*B* = .94, *SE* = .47, *p* = .046).

In summary, the more analytical words a medical YouTuber employed, the more views the video received. Conversely, the more positive emotion or anger a medical YouTuber expressed, the fewer views the video received. As for the number of likes and comments, among all independent variables, anxiety is the only variable significantly associated with either of the two dependent variables. Put differently, as a medical YouTuber showed more anxiety, the video received a greater number of likes and comments.

### Emotional alignment between medical YouTubers and viewers

The multiple linear regression analysis for “positive emotion” (RQ4-1) displayed that *viewers’ positive emotion* was positively associated with *medical YouTubers’ positive emotion* (*B* = 1.67, *SE* = .20, *p* < .001; see Table 3). Likewise, the multiple linear regression analysis for “negative emotion” (RQ4-2) showed that *viewers’ negative emotion* was also positively associated with *medical YouTubers’ negative emotion* (*B* = .43, *SE* = .08, *p* < .001). The *R*^2^ values in Table 3 show that the two regression models predicting viewers’ positive and negative emotions can explain 37% and 34% of the variances in the two dependent variables, respectively. Taken together, regardless of emotion type, the viewers’ emotion is positively correlated with the medical YouTubers’ emotion.

**Table 3:** OLS regression models predicting emotions of medical YouTubers’ viewers.

		Viewers’ positive emotion	Viewers’ negative emotion
		*B* (*SE*)	*B* (*SE*)
**Predictors** ^**a**^	Medical YouTuber’s positive emotion	1.67*** (.20)	.10 (.07)
	Medical YouTuber’s negative emotion	.09 (.21)	.43*** (.08)
**Control variables**	Number of comments^b^	-.79*** (.19)	.36*** (.07)
	Number of words per comment^c^	-2.38** (.83)	.18 (.30)
**Constant**		2.28*** (.32)	.47*** (.12)
**Adjusted *R*** ^**2**^		.37	.34

^a^When the independent variable was *medical YouTuber’s positive emotion*, *medical YouTuber’s negative emotion* functioned as a control variable, and vice versa.

^b^The scale of *number of comments* was adjusted by multiplying .0001 for both of the models.

^c^The scale of *number of words per comment* was adjusted by multiplying .01 for both of the models.

** *p* < .01

*** *p* < .001.

## Discussion

This study observed medical YouTubers’ COVID-related videos and their viewers’ comments to better understand science communication on social media. Specifically, this study explored how medical YouTubers’ languages related to analytical thinking (RQ1), positive emotion (RQ2), and negative emotion (RQ3) are associated with their viewers’ engagement. In addition, we tested if there is an emotional alignment between medical YouTubers and their viewers (RQ4). The present study produced novel findings that have not been previously suggested, which we will discuss in this section along with their practical and theoretical implications. Furthermore, the results of this study denote the important differences between the social media engagement metrics—number of views, likes, and comments—which will also be elucidated in this section.

### Medical YouTubers’ analytical words (RQ1)

Our results about analytical words presented interesting findings. Medical YouTubers’ analytical words were positively associated with the number of views. In other words, viewers paid more attention to information-oriented videos with analytical words. This association corresponds to the information seeking behavior described by the risk information seeking and processing (RISP) model in risk communication research [63,64]. RISP suggests that people try to assess risk during a hazard. Likewise, YouTube users probably wanted to watch videos with an in-depth analysis of the life-threatening virus or find preventive measures during the pandemic, where the YouTubers naturally used more analytical words. Moreover, RISP elucidates that people seek channels providing high-quality information to prepare for risks. Accordingly, users might be drawn to the channels of medical YouTubers, who have reliable expertise through their licenses or training. Our findings align with empirical studies on risk communication, particularly during the COVID-19 pandemic, which found that social media was a crucial information source [e.g., 65].

That being said, medical YouTubers’ analytical thinking did not have a significant association with the number of either likes or comments. Put differently, although medical YouTubers’ analytical words succeeded in attracting users to their videos, it did not significantly lead to those users’ support or participation in the comment threads. These findings correspond to prior literature on science communication. Brossard [9] suggests that online environments have changed how scientists need to communicate with non-experts, and the current study demonstrates that scientists’ analytical words, which were typical of traditional science communication, are not necessarily conducive to increased viewer engagement in the current study. In fact, our findings about medical YouTubers’ analytical language may seem confusing to scientists who seek to communicate with the public on social media. In a case like the COVID-19 pandemic, in which misinformation is a serious issue, scientists need to convey credible information based on their scientific knowledge, which makes them likely to entail analytical words. However, it turns out that those words are not necessarily helpful for some types of viewer engagement.

Here, we suggest some practical suggestions based on the findings of this study. First of all, it is essential for scientists to determine the goals of their activities (e.g., increasing awareness, changing behavior, sharing knowledge) as discussed by Besley and Dudo [66]. Then, if the goal of science communication is to gain more attention, scientists should use analytical words as needed. Our results showed that use of words related to analytical thinking significantly increases viewers’ attention. Meanwhile, if the goal of science communication is to obtain support for the scientists’ ideas or to hear viewers’ opinions, scientists do not need to employ complicated or analytical words.

To illustrate with the COVID-19 case, the main purpose of most medical YouTubers’ COVID-related videos in the early pandemic was to inform their viewers of critical information about the virus, rather than asking them for support or comments. Thus, scientists’ use of analytical language was appropriate in this emergent and awareness-necessary situation. Indeed, the use of analytical language helped establish the boundary work [67], demonstrating scientists’ authority and expertise during the crisis. That said, the use of analytical language might not be as helpful towards the end of the pandemic, given the change in urgency. To summarize, scientists should vary the extent of their analytical language in science communication with the public, depending on their main purpose of communication.

### Medical YouTubers’ emotion (RQ2 & RQ3)

This study found that positive emotion was negatively associated with the number of views, indicating viewers’ tendency to avoid COVID-related videos where the YouTubers spoke in a positive way. Given that LIWC counts only words clearly expressing positive emotion for this variable, the results are commonsensical; viewers might consider explicitly positive COVID-related videos tone-deaf or oblivious to the seriousness of the situation at the time, when the death toll was soaring. It is worth noting that our findings contrast with prior literature on general science education, which suggests that positive emotion can potentially help increase learners’ interests and, consequently, their attention while learning science in general [31]. This difference likely originates from the unique context of COVID-19. The seriousness of the pandemic is distinguishable from other general science/health education cases; that is to say, context matters. Meanwhile, positive emotion has no significant association with both the number of likes and comments. This may denote that, as with analytical thinking, positive emotion functions as a crucial factor for social media users’ attention, but not for their further engagement, like support or participation in the discussion.

Regarding the three types of negative emotions—anger, anxiety, and sadness—their associations with the engagement metrics considerably varied: anger was negatively associated with the number of views, anxiety was positively associated with both the number of likes and comments, and sadness was not significantly associated with any of the three metrics. The negative association between anger and the number of views was interesting, especially given that anger was the only negative emotion correlated with the number of views and that it was not significantly associated with the other two engagement metrics. This perhaps implies that people did not expect emotional explosions from medical YouTubers’ videos on COVID-19, but organized information based on their professional analyses. In fact, this finding does not coincide with prior literature on anger as Parkinson [68] suggests that “‘anger’ serves to draw others’ attention.” Since expressing anger may not appear scientific or trustworthy and can seem overly emotional, it is possible that the information-seeking public during the pandemic was less likely to watch videos conveying anger, which might attract viewers during non-urgent times.

When it comes to anxiety, this variable was significantly associated with the number of likes and comments, but not with the number of views, which is exactly the opposite result of anger. When a medical YouTuber used more words related to anxiety, viewers were more likely to support the video and write a comment in the comment section. It is worth highlighting that anxiety is the only variable of all predictors that has a correlation with the number of either likes or comments. This suggests that medical YouTubers’ anxiety was the key emotion that significantly related to viewers’ support and participation in the discussion in this study, whereas other emotions did not significantly associate with deeper viewer engagement beyond attention. The importance of anxiety is understandable considering relevant literature and the nature of COVID-19. Izard [69] suggests that anxiety is an anticipatory response to an unfortunate event that may occur in the future. Obviously, the outbreak of COVID-19 was an extremely unfortunate event that made people feel anxious even before it was widespread. Given this prevalent anxiety during the pandemic, medical YouTubers’ anxiety was likely one of the emotions that users most resonated with [70], which probably led to the significant engagement found. This finding is also in line with previous literature on science communication, which emphasizes the importance of *vulnerability* [24].

To summarize, our findings exhibit that scientists’ discrete emotions can work distinctively on their science communication with the public, which corresponds to recent literature on social media communication during the pandemic [49]. Future research could further investigate this topic by employing other research methods, notably experiment, to address the reasons behind the different functions of emotions in user engagement and the relationships between those emotions.

### Emotional alignment between medical YouTubers and viewers (RQ4)

Our results about emotional alignment between medical YouTubers and their viewers showed that both positive and negative emotions are positively correlated. These findings support prior literature highlighting the importance of emotion in science communication [2,26] and demonstrate that scientists and the audience can emotionally engage with each other via social media. It is somewhat surprising that medical YouTubers’ emotion in their COVID-related videos, which are likely less emotional than videos about other topics, is still essential for connecting with viewers. In fact, regardless of whether the emotion is positive or negative, viewers’ emotional alignment with the YouTubers could essentially be empathy, the importance of which prior literature on crisis events highlights [71]. Our findings suggest the possibility that, as with some other crisis events, the COVID-19 pandemic gave rise to empathy among people, even in online spaces.

In addition, this study supports some relevant theoretical notions including emotional contagion [54,72] and homophily [73]. Our findings align with previous studies using these two concepts as theoretical frameworks, especially those about YouTube; Rosenbusch et al.’s study [58] about 2,083 YouTube vlogs and their comments demonstrates the presences of both emotional contagion and homophily through multilevel analysis [58]; likewise, Ladhari et al. [74] found that the homophily between YouTubers and their viewers increases the YouTubers’ popularity. Although the current study is not specifically designed to test emotional contagion or homophily, we hope that future research in those research avenues could utilize our findings as a valuable clue.

### Social media user engagement metrics

While it was not the initial focus of this study, the difference found between the number of views and the number of likes and comments is another meaningful finding. Our regression models displayed that *anxiety* was the only independent variable that was significantly associated with the number of likes and comments. Meanwhile, *analytical thinking*, *positive emotion*, and *anger* were significantly associated with the number of views. These results provide a valuable message for social media research in general; there is a significant boundary between the public’s “attention” and “engagement.” This study utilized view count as one of the metrics representing the extent of viewers’ engagement based on prior literature [7,38]. However, our results reveal the possibility that view count cannot accurately represent engagement. In fact, social media researchers are mixed about the validity of view count as an engagement metric [75]; some studies exclude view count and only include the number of likes and comments when measuring social media engagement [76,77]. For instance, in Kim’s paper about social media engagement metrics in risk communication [77], the author reviews the meanings of view, like, and comment counts based on prior literature and excludes view count when measuring social media engagement.

Heldman et al.’s paper [78], one of the earliest papers to explore social media engagement and health communication, bespeaks why we need to distinguish social media users’ attention and engagement and to exclude view count from engagement metrics. In the paper, the authors define the concept of social media engagement as “a multi-way interaction between and among an organization and digital communities that could take many forms, using social media channels to facilitate that interaction” [78]. One clear keyword in this definition is “interaction.” Heldman et al. as well as a host of other researchers [e.g., 79,80] consider the most important feature of social media to be multi-way interaction and view social media engagement as a result of these interactions. From this perspective, the number of likes and comments can function as engagement metrics since both can be considered a type of interaction (i.e., viewer feedback). Clicking “like” sends an implicit indication of support to the medical YouTuber, while comments are more explicit messages. Meanwhile, the number of views does not represent any interaction type, but rather serves as a simple record of users’ attention to the video. Particularly concerning YouTube, every time a video is played for at least 30 seconds, the platform automatically increments the view count; thus, the number can include many incomplete views. Taken together, while there exist studies that tried to gauge users’ engagement with the number of views [7,38] including the current study, we need to consider distinguishing the concept of attention from engagement.

Furthermore, the results of this study raise a methodological question about the effectiveness of using the number of views as a dependent variable, specifically when YouTubers’ language is the independent variable. On YouTube, a view is counted at the moment when a user watches a video for 30 seconds, which means that the view count likely occurs *before* the user has fully watched the video. In contrast, users typically click the like button or write a comment *after* watching the video as much as they want. Thus, the only few cases that YouTubers’ language can influence the number of views are (1) when the viewer watches the same video again or recommends the video to others influenced by the YouTuber’s language, or (2) when the viewer stops the watch before reaching the minimum required time (30 seconds) for a view count due to the YouTuber’s language. Given that these cases may happen relatively rarely, we suggest that future research on YouTube should not use the number of views as an engagement metric when testing the effect or association of YouTubers’ language.

Beyond this issue, some recent studies on science communication employ novel engagement metrics to better measure user engagement. Yang et al. [81] introduced additional measures for social media engagement, including average view duration, average percentage viewed, number of subscribers gained, and number of playlists added in, alongside more typical social media engagement measures, including number of views and comments. Their use of this wide array of variables was enabled by partnering with a certain organization, the American Chemical Society. Following this study, future research could incorporate diverse dependent variables through collaboration with relevant organizations. Furthermore, building on the present study, researchers could explore viewers’ text as a source for measuring specific aspects of user engagement, thereby diversifying independent variables as well.

### Limitations and future research

Though this study has several strengths, it has some notable limitations, as with any study. First, our sample is from only five YouTube channels. While we tried to find more medical YouTubers to generalize our findings, we had to maintain our rigid sampling criteria, such as YouTubers with an official license/degree about medicine/health, for reliable results. Due to this limited sample size, our data were collected only from male medical YouTubers’ channels. As previous studies show an association between gender and language [82], there is a possibility that this limitation influenced our results. Additionally, our sample focuses on COVID-19; as such, it is probable that some of our findings may not extrapolate to other contexts of science communication. We hope that future research can extend our findings with a larger sample, including posts about a variety of topics by scientists of diverse backgrounds.

Another limitation of this study is its method, NLP. While LIWC is known as a reliable NLP software [49,61], its ability to sense human emotion is not flawless. To be specific, since the mechanism of LIWC is based on predetermined dictionaries, it cannot flexibly understand atypical meanings of words, including sarcasm or online slang. Therefore, our results based on LIWC scores should be interpreted with caution. We hope that future research can overcome this limitation with an advanced NLP method. In addition, the use of LIWC was accompanied by some particular limitations. The software does not allow us to measure any specific types of positive emotion. Similarly, it supports only three types of negative emotion—anger, anxiety, and sadness—which were utilized in this study. However, it is clear that in reality, there are multiple types of both positive emotion and negative emotion. We suggest that future work in this field try to explore a wider range of emotion types using novel research tools. It is particularly noteworthy that, if the negative emotion type “fear” can be measured in a reliable way, future research could further investigate how fear appeal, a recurring topic in health/science communication, operates during health crises such as the COVID-19 pandemic.

Finally, the original aim of the study was to identify *general* patterns of the three types of user engagement on the selected YouTube videos. However, not all social media users have the same background and preferences, and some of them do not show their engagement through the three engagement proxies in this study. In order to observe social media users’ more nuanced engagement, incorporating additional back-end data in collaboration with YouTubers may be useful, as Yang et al. [81] indicated. Moreover, conducting interviews with non-experts would be fruitful, as Hill et al. [14] interviewed science communicators on YouTube to gain their insights. This is the next step in our research that we plan to pursue.

Despite these limitations, the current study identified new insights into interactive science communication on YouTube. We hope that this study can be a valuable step towards future research on diverse aspects of science communication through video-based social media platforms.
